# Exogenously Applied Trehalose Augments Cadmium Stress Tolerance and Yield of Mung Bean (*Vigna radiata* L.) Grown in Soil and Hydroponic Systems through Reducing Cd Uptake and Enhancing Photosynthetic Efficiency and Antioxidant Defense Systems

**DOI:** 10.3390/plants11060822

**Published:** 2022-03-19

**Authors:** Sadia Rehman, Muhammad Umer Chattha, Imran Khan, Athar Mahmood, Muhammad Umair Hassan, Asma A. Al-Huqail, Mohamed Z. M. Salem, Hayssam M. Ali, Christophe Hano, Mohamed A. El-Esawi

**Affiliations:** 1 Department of Botany, University of Agriculture, Faisalabad 38040, Pakistan; sadiakalyar01@gmail.com; 2 Department of Agronomy, University of Agriculture, Faisalabad 38040, Pakistan; drumer@uaf.edu.pk (M.U.C.); drimran@uaf.edu.pk (I.K.); athar.mahmood@uaf.edu.pk (A.M.); 3 Research Center on Ecological Sciences, Jiangxi Agricultural University, Nanchang 330045, China; muhassanuaf@gmail.com; 4 Botany and Microbiology Department, College of Science, King Saud University, P.O. Box 2455, Riyadh 11451, Saudi Arabia; aalhuqail@ksu.edu.sa (A.A.A.-H.); hayhassan@ksu.edu.sa (H.M.A.); 5 Forestry and Wood Technology Department, Faculty of Agriculture (El-Shatby), Alexandria University, Alexandria 21545, Egypt; mohamed-salem@alexu.edu.eg; 6 Laboratoire de Biologie des Ligneux et des Grandes Cultures (LBLGC), INRAE USC1328, Université d’Orléans, 28000 Chartres, France; hano@univ-orleans.fr; 7 Botany Department, Faculty of Science, Tanta University, Tanta 31527, Egypt

**Keywords:** cadmium, mung bean, photosynthetic pigments, antioxidants, ROS, trehalose

## Abstract

Cadmium (Cd) toxicity is a serious environmental issue causing a significant reduction in crop growth and productivity globally. Trehalose (Tre) has emerged as an important reducing sugar that can reduce the adverse impacts of different abiotic stresses. Therefore, the present investigation was performed to determine the key role of Tre in alleviating Cd stress in the mung bean (*Vigna radiata* L.) crop. The study was comprised of different treatments of cadmium (0, 10, 20 mg kg^−1^ soil) and Tre (0, 15 and 30 mM). Cd stress significantly restricted the growth and yield of mung bean. However, Tre supplementation markedly improved growth and yield due to pronounced reductions in Cd uptake and Cd-induced oxidative stress as shown by the lower production of hydrogen peroxide (H_2_O_2_), electrolyte leakage (EL) and malondialdehyde (MDA) in Cd-stressed plants as well as by the enhanced activities of antioxidant enzymes (CAT, POD, APX and AsA). Moreover, the ameliorative role of Tre to Cd toxicity was also demonstrated by its ability to enhance chlorophyll contents, total soluble protein (TSP) and free amino acids (FAA). Taken together, Tre supplementation played a key beneficial role in improving Cd stress tolerance and yield traits of mung bean through restricting Cd uptake and enhancing photosynthetic capacity, osmolytes biosynthesis and antioxidant activities.

## 1. Introduction

Environmental pollution and ecological damages have been substantially increased in recent time owing to rapid industrialization. Heavy metal pollution in the soil has become a serious environmental issue nowadays and it negatively affects food production and human health [1]. Cadmium (Cd) is a non-essential and toxic element [2] readily absorbed by plant roots, causing serious structural and functional alternations as well as inhibition of the seed germination and root growth [3]. Cadmium inhibits the various physiological processes including photosynthesis, respiration, water movement, leaf gas exchange and, therefore, impairs plant metabolism [4,5,6,7]. Additionally, Cd stress reduces the chlorophyll synthesis [8] and induces antioxidant activities by increasing the production of reactive oxygen species (ROS). Cd stress also decreases plant biomass production by inducing oxidative damage and decreasing the nutrient uptake and photosynthetic processes [9,10,11]. Moreover, Cd stress negatively affects the mineral uptake, transpiration rate and stomatal conductance [12]. Cd-induced injury is related to oxidative stress which causes damages to protein, DNA and lipids [13,14,15,16,17,18], and eventually leads to plant death [10]. However, plants respond to that and reduce the damages of heavy metals stress through activating their diverse enzymatic and non-enzymatic anti-oxidants and the accumulation of various osmolytes [10]. Cd easily accumulates in plant organs and in turn enters into the human food chain and causes chronic diseases [19]. Therefore, it is urgently need to find appropriate strategies to remediate the Cd contaminated soils to prevent its effects on plants and human health.

Globally, different techniques including leaching, stabilization and phytoremediation are used to remediate the Cd contaminated soils to prevent its impacts on the environment and humans [20,21]. Nonetheless, the current remediation practices to treat heavy metal-polluted soils have shown promising results; however, they are expensive [22]. Likewise, the use of different chelating agents to treat the heavy metal-polluted soils is also expensive and they also cause pollution owing to their artificial footprints and non-biodegradability [23]. Thus, to address these concerns the application of different osmo-protectants is suggested as an imperative strategy to ensure safe production and improve the plant tolerance against different abiotic stresses. Different protectants including proline, glycine betaine (GB) and trehalose achieved global attention due to their excellent efficiency in protecting plants from the deleterious effects of different stresses [24,25,26,27]. The selection of a suitable osmo-protectant is crucial to increase the plant’s ability to cope with stresses. In this context, it is reported that exogenously applied osmo-protectants protect plants from the adverse effects of heavy metal stress [8].

Trehalose (Tre) is a non-reducing sugar and it is considered as an important osmo- protectant against different stresses [28,29]. Tre formation in plants involves the production of trehalose-6-phosphate (T6P) from glucose-6-phosphate and UDP-glucose by trehalose-6-phosphate synthase (TPS), and the subsequent dephosphorylation of T6P to Tre by trehalose-6-phosphate phosphatase (TPP) [30]. Two molecules of uridine diphosphate glucose (UDP-Glc) and glucose-6-phosphate (Glc-6-P) are used for the biosynthesis of Tre in plants. The enzyme TSP catalyzed UDP-Glc and Glc-6-P into T6P [31,32] whereas enzyme trehalose-6-phosphate phosphatase (TPP) catalyzed the T6P into Tre as a final product [33,34]. Tre is an important energy source and possesses special physio-chemical characteristics (glycosidic bond and higher hydrophilicity); therefore, it stabilizes dehydrated proteins, lipids and membranes and protects the biological structure from oxidative damages [23]. Trehalose acts as an imperious elicitor of the genes involved in stress responses and ROS detoxification [35]. Nonetheless, endogenous Tre production in most plants is not adequate to mitigate the effects of different stresses, therefore, in this case exogenously applied Tre increased the endogenous Tre levels and was recommended as an important alternate strategy to induce stress tolerance [36]. The exogenous Tre application alters the enzymatic activities and, therefore, reduces the ROS level [37]. Exogenously applied Tre increased the biomass production under salty conditions by reducing H_2_O_2_ and MDA accumulation [35]. Moreover, Tre application increased the activities of the anti-oxidants enzymes (CAT, SOD, POD) and the internal trehalose content in rice plants grown in hydroponic culture under Cd stress [23]. Moreover, formation of Cd–Tre chelation effectively reduces the Cd mobility and toxicity to rice plant organs and therefore improves rice growth under Cd stress [23].

Mung bean (*Vigna radiata* L.) is an important annual crop cultivated globally as grain, vegetable and livestock feed and it is also used for medicinal purposes [38,39]. Mung bean is considered to be sensitive to Cd stress; therefore, Cd stress can cause significant yield losses in mung bean. Limited information is available about the mechanistic role of Tre in mitigating the deleterious impacts of Cd stress. Thus, this research was carried out to investigate and assess the impacts of exogenously applied Tre on the growth, physiological attributes and antioxidant systems of mung bean plants grown under Cd stress conditions. The present study provides interesting insights into the mechanistic role of Tre in improving Cd stress tolerance and yield of mung bean.

## 2. Materials and Methods

### 2.1. Plant Material and Growth Conditions

The present study was conducted to determine the effect of trehalose (Tre) on mung bean (*Vigna radiata* L.) plants grown under cadmium stress. This experimental trial was carried out in pots containing soil collected from the upper soil layer (1–2 cm) from previously grown rice crop. The soil was sandy loam having pH of 7.82, organic matter of 0.82%, nitrogen of 0.042%, available phosphorus of 6.65 ppm and potassium of 160 ppm. The fertilizers di-ammonium phosphate (5.50 g) and sulfate of potash (1.82 g) were applied to each pot to fulfill nutrient needs. Silt and soil were mixed thoroughly (1:2) and pots contained 6 kg soil and silt. The study was comprised of different Cd stress levels, i.e., control (no Cd), 10 and 20 mg kg^−1^ soil and Tre application levels, i.e., control, 15 and 30 mM. Cadmium chloride was used as the cadmium source, and it was thoroughly mixed into the soil. Seeds of Azri-Mung-2006 were obtained from the Ayub Agricultural Research Institute (AARI) Faisalabad and used in the present study. Healthy seeds were sterilized by soaking in 5% sodium hypochlorite solution for 5 minutes and then carefully washed 2–3 times with water. Afterwards, 10 seeds were sown in every pot at 1 cm depth. After 15 days of germination, weeding and thinning were completed to maintain uniform and healthy seedlings (6 plants) in each pot. Moreover, pots were visited regularly and irrigation water was applied to pots according to the crop requirement on the basis of visual experience. Before flowering, the mung bean plants were subjected to the foliar application of Tre (0, 15 and 30 mM). The foliar application of Tre was completed only once using a hand sprayer. The plants were collected at the podding stage and used for the subsequent experimental analyses.

A hydroponic experiment has also been carried out to estimate the morphological and growth traits of mung bean plants grown under natural and Cd stress conditions in order to validate and compare the recorded morphological data with those recorded for plants grown in the above-mentioned soil. The sterilized mung bean seeds were allowed to grow on wet filter papers at 23 °C for 7 days. The germinated mung bean seedlings were then transferred into hydroponic plastic pots, containing Hoagland plant nutrient solution and left to grow in a growth chamber having constant conditions (27/20 °C (day/night) and 70% humidity). At the third leaf stage, uniform seedlings were selected and treated in solutions containing diverse levels of Cd and Tre for four days. The concentrations of Cd and Tre used to treat mung bean plants were as follows: Cd (0, 200, and 400 μM) and Tre (0, 15 and 30 mM). Following treatment for four days, the seedlings were harvested and used for measuring their morphological traits.

### 2.2. Growth Traits

Three plants per soil and hydroponic pots were used for growth traits’ measurement. Roots and shoots were separated and their lengths were measured. Roots and shoots were also weighed to determine their fresh weights. Roots and shoots were then oven-dried (72 °C) for three hours to determine their dry weights. Moreover, the number of leaves per plant were counted and averaged.

### 2.3. Measurement of Photosynthetic Pigments Contents in Soil-Grown Plants

The standard procedure of Arnon [40] was used to determine the contents of photosynthetic pigments in soil-grown plants. Approximately 0.5 g of plant samples were taken and ground in 5 mL of methanol solution for 24 h. The mixture was then centrifuged for 10 min at 10,000 rpm. The absorbance of the extract was then recorded at 663, 645 and 480 nm to determine chlorophyll (a, b) and carotenoid contents. The following standard formula were used to compute the levels of chlorophylls (a, b) and carotenoids:

chlorophyll a=(12.7 (OD663)−2.69 (OD645))×V/1000×W

chlorophyll b=(22.9 (OD645)−4.68 (OD663))×V/1000×W

Carotenoid=[(OD480)+0.114 (OD663)−0.638 (OD645)]/2500

where *V* is the volume of sample supernatant and *W* is the weight of the sample.

For the determination of anthocyanin content, 0.5 g of fresh leaves’ sample was homogenized in potassium buffer (10 mL). The mixture was then centrifuged for 15 min at 15,000 rpm. The absorbance of the supernatant was recorded at 600 nm to determine anthocyanin content.

### 2.4. Determination of Relative Water Content (RWC) in Soil-Grown Plants

For the measurement of RWC in soil-grown plants, the standard protocol of Mostofa and Fujita [41] was used. The second leaf was plucked from different mung bean seedlings and weighed to determine the fresh weight (FW). The leaves were dipped in distilled water and kept in the dark for 24 h. Leaves were left in the air to dry and then weighed to determine their turgid weight. Samples were then oven-dried (70 °C) for two hours to determine dry weight. RWC was then estimated using the following standard formula:

$$\mathrm{RWC}\left(\%\right)=\frac{\left(\mathrm{FW}-\mathrm{DW}\right)}{\left(\mathrm{TW}-\mathrm{DW}\right)}\times 100$$

where FW is the fresh weight, DW is the dry weight, and TW is the turgid weight.

### 2.5. Determination of Electrolyte Leakage (EL), Malondialdehyde (MDA) and Hydrogen Peroxide (H_2_O_2_) Contents in Soil-Grown Plants

To estimate the electrolyte leakage (*EL*) in soil-grown plants, fresh leaves were collected from each treatment and washed carefully with distilled water. Approximately 0.5 g of leaf sample was cut into pieces and placed in a test tube having distilled water (50 mL) and then first electrical conductivity (EC_1_) was read on EC meter after 3 h. Afterwards, test tubes were incubated at 120 °C for 20 min and the second value of EC_2_ was recorded. EL was estimated using the following standard formula:

*EL* (%) = (EC_1_ ÷ EC_2_) × 100

For determination of the MDA content in soil-grown plants, 0.5 g mung bean leaves were homogenized in 5 mL of 5% TCA. The mixture was then centrifuged for 15 min at 15,000 rpm and the supernatant was collected. A total of 1 mL TCA (0.5%) and 1 mL of TBA (thiobutyric acid: 20%) were then added into the supernatant and placed at 90 °C for 50 min. Absorbance was noted at 532 nm and 600 nm to determine MDA content following Cakmak and Horst [42]. For measuring the H_2_O_2_ content in soil-grown plants, 0.25 g of plant leaves were ground in 5 mL of (0.1%) *w*/*v* trichloroacetic acid (TCA) using a pestle and mortar under chilled conditions. The extract was then centrifuged at 15,000 rpm at 4 °C for 10 min and the supernatant was collected. Approximately, 1 mL of supernatant, 100 µL potassium phosphate buffer (pH 7.0) and 1 mL of 1 M potassium iodide were mixed well and the absorbance was recorded at 390 nm following Velikova et al. [43].

### 2.6. Determination of the Total Soluble Proteins (TSP) and Free Amino Acids (FAA) in Soil-Grown Plants

Plant leaf samples (0.5 g) were homogenized in 5 mL potassium phosphate (50 mM). The mixture was then centrifuged at 12,000 rpm for 15 min. A mixture of 100 µL of fresh plant extract and 100 µL of Bradford reagent were then prepared. Protein intensity in leaf tissue was spectrophotometrically measured at 595 nm following Bradford [44]. Moreover, the protocol of the Van Slyke technique [45] was followed to estimate the FAA content of mung bean plants. Fresh leaves (0.5 g) of mung bean were ground in 5 mL potassium phosphate buffer (50 mM) in an ice bath. The homogenate was then centrifuged at 15,000 rpm for 15 min at 4 °C. Plant extract was then treated with 1 mL of ninhydrine (2%) and pyridine (10%) solutions in a test tube. The samples were then heated up at 90 °C for 30 min. After heating, the volume of the mixture was brought up to 20 mL and absorbance was noted at 570 nm.

### 2.7. Antioxidant Activities Assay in Soil-Grown Plants

To analyze the catalase (CAT) activity of mung bean plants, the procedure of Chance and Maehly [46] was followed. Plant material (0.5 g) was ground in 5 mL of potassium buffer (50 mM). The mixture was then centrifuged at 4 °C and 10,000 rpm for 15 min. Approximately, 2.5 mL of potassium phosphate buffer was added into a test tube containing 100 µL of H_2_O_2_. After that, 100 µL of plant crude extract was rapidly added into the reaction mixture and absorbance was noted at 240 nm. Peroxidase (POD) activity was estimated following the method described by Guan et al. [47]. Approximately, 0.5 g of leaf sample was ground in 5 mL potassium buffer (50 mM) under ice cold conditions and centrifuged for 15 min at 15,000 rpm and the supernatant was then collected. POD reaction contained 100 µL of H_2_O_2_, 100 µL guaiacol and 100 µL of enzyme extract, and the absorbance was then read at 470 nm. Ascorbate peroxidase (APX) activity was also assessed following the method of Nakano and Asada [48]. Approximately, 0.5 g of plant samples were ground into 5 mL of potassium buffer (50 mL) and centrifuged for 15 min at 15,000 rpm to collect the supernatant. The reaction medium contained 600 µL H_2_O_2_, 100 µL ascorbic acid, 1 mL potassium buffer and 100 µL of enzyme extract. Absorbance was then noted at 290 nm. The method of Mukherjee and Choudhri [49] was used to assess the ascorbic acid activity. An amount of 0.5 g of plant leaves were ground in 5 mL of 10 % trichloroacetic acid. The mixture was then centrifuged at 15,000 rpm for 15 min at 4 °C and was then kept for 30 min at 30 °C and absorbance was noted at 520 mM to determine ascorbic acid content.

### 2.8. Determination of Yield Components in Soil-Grown Plants

Plants’ pods were collected and counted and their lengths were measured. Plants were then harvested to determine grains’ yield and 100 grain weight.

### 2.9. Determination of Cadmium Concentration in Organs of Soil-Grown Plants

The plant samples (roots, stems, leaves and grains) were collected, dried and stored. Afterward, 0.5 g of each plant was ground into powder and digested by adding HNO_3_: HClO_4_ in 2:1 ratio [50]. After digestion, the concentration of Cd in plant organs was measured using atomic absorption spectrometry and calculated following the formula: Cd concentration = (reading of AAS × dilution factor)/dry weight of root/shoot/seed and expressed in µg g^−1^ of D.M.

### 2.10. Statistical Analysis

The collected data were subjected to ANOVA using Statistix 8.1 software and the significant difference among means was computed by LSD test (*p* < 0.05) [51]. Moreover, sigma-plot 10 was used to prepare graphs.

## 3. Results

### 3.1. Growth Traits

The findings indicated that the different levels of Tre application and Cd stress had significant impacts on the growth traits of mung bean plants grown in soil and hydroponic systems (Table 1 and Table 2). Cd stress significantly decreased the growth and biomass production; however, it was dose-dependent and a higher Cd concentration caused more reduction as compared to a lower dose of Cd stress. Nonetheless, Tre application induced a markedly increase in both growth and biomass production under control and Cd stress conditions (Table 1 and Table 2). The highest RL (6.67 cm) and SL (28.10) were noted under no Cd and 30 mM Tre, and the lowest RL (3.17 cm) and SL (19.20 cm) was noted under highest Cd level and without Tre supplementation (Table 1). Cd stress significantly reduced the RFW, SFW, RDW and SDW of mung bean plants (Table 1 and Table 2). Nonetheless, Tre application (30 mM) significantly increased the RFW, SFW, RDW and SDW under Cd stress and control conditions (Table 1 and Table 2). Likewise, a decrease in the number of LPP was recorded under both Cd stress levels, whereas Tre application induced a significant increase in LPP under control and both Cd stress levels (Table 1). The application of 30 mM Tre significantly increased the number of LPP as compared to control and 15 mM Tre application under control and Cd stress (10 and 20 mg kg^−1^) (Table 1).

### 3.2. Photosynthetic Pigments and Anthocyanin Contents

**Figure 1:**
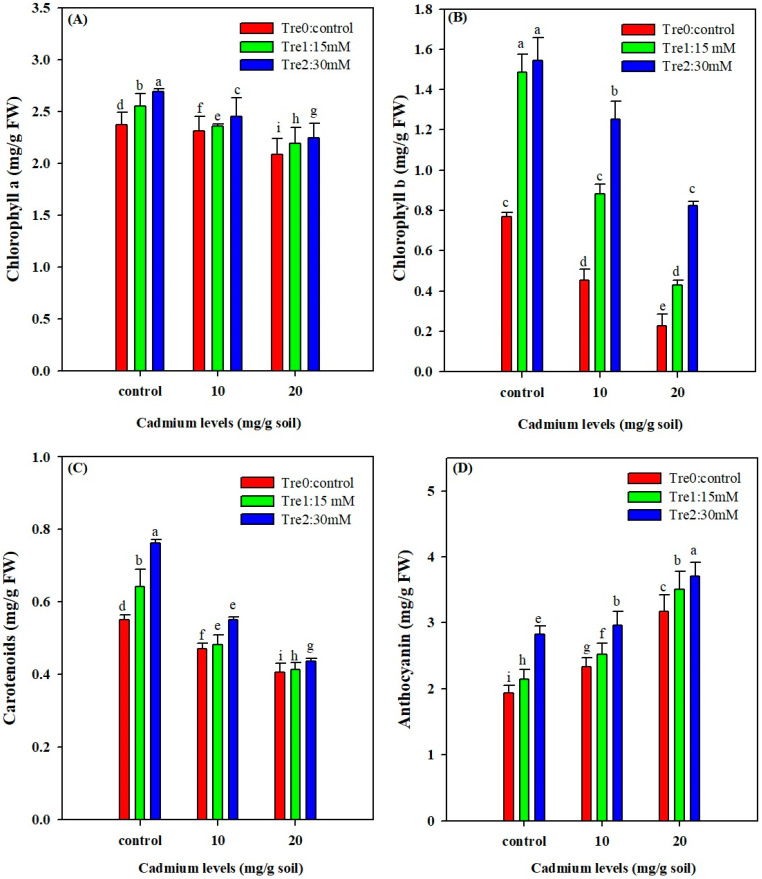
Effect of foliar application of Tre on chlorophyll a (**A**); chlorophyll b (**B**); carotenoid (**C**); and anthocyanin (**D**) contents of mung bean plants grown under Cd stress. The values given in vertical bars are showing the mean of three replicates with ± S.E and the different letters above the bars under the same treatment show the significant differences at *p* < 0.05.

Cd stress significantly reduced the biosynthesis of photosynthetic pigments (chlorophyll and carotenoid) of mung bean plants. Conversely, Tre application significantly increased chlorophyll and carotenoid contents under control and Cd stress. The highest foliar spray of Tre (30 mM) significantly increased chlorophyll and carotenoid contents under Cd stress (Figure 1). Cd stress also significantly reduced the carotenoid and anthocyanin contents (Figure 1). Nonetheless, Tre application (30 mM) improved carotenoid contents by 22% and 55%, and anthocyanin contents by 13% and 50% under Cd stress (10 and 20 mg kg^−1^) (Figure 1).

### 3.3. Relative Water Content

**Figure 2:**
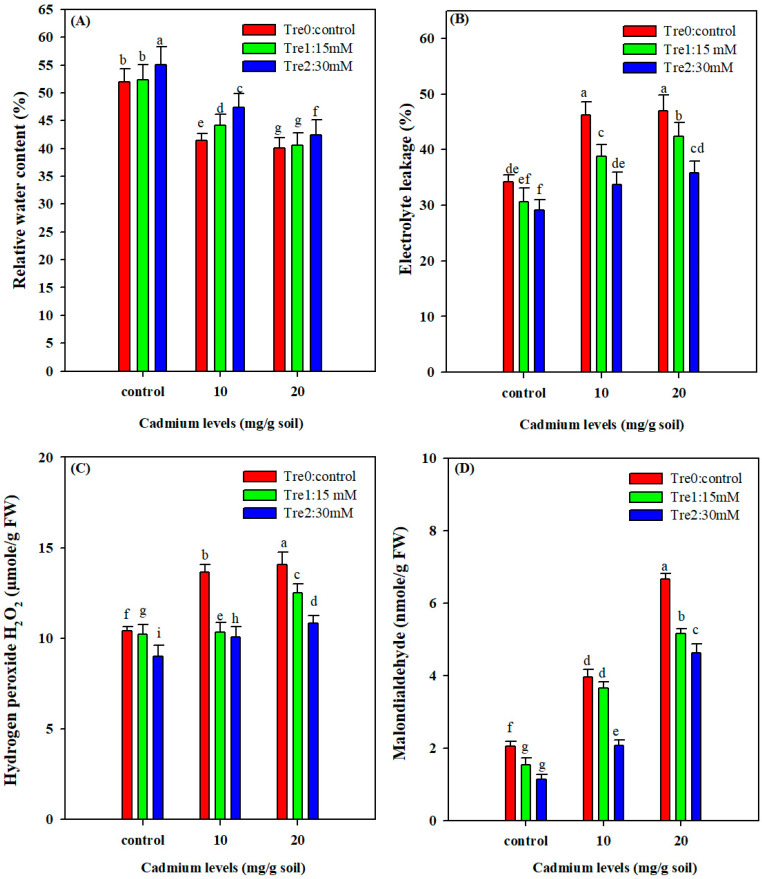
Effect of foliar application of Tre on RWC (**A**); electrolyte leakage (**B**); H_2_O_2_ (**C**); and MDA (**D**) contents of mung bean grown under Cd stress. The values are the means of three replicates with ± S.E and the different letters above the bars under the same treatment show the significant differences at *p* < 0.05.

The RWC was significantly reduced under Cd stress. However, the foliar application of Tre markedly improved the RWC under normal and Cd stress conditions. Both levels of Cd stress reduced the RWC and the maximum decrease was recorded under 20 mg kg^−1^ Cd stress (Figure 2). On the other hand, Tre application (30 mM) enhanced the RWC of mung bean plants by 3% and 9% at the lowest (10 mg kg^−1^) and the highest (20 mg kg^−1^) Cd stress levels (Figure 2).

### 3.4. Electrolyte Leakage, MDA and H_2_O_2_

Electrolyte leakage (EL) showed a significant increase under Cd stress conditions (Figure 2). The maximum EL was recorded in the highest Cd (20 mg kg^−1^) level without Tre application and the lowest EL was recorded in control (no Cd) with Tre application of 30 mM (Figure 2). As such, Tre supplementation significantly reduced the EL (Figure 2). Foliar spray of Tre (30 mM) minimized the negative effects of EL and reduced the EL by 14% and 29% under the 10 and 20 mg kg^−1^ Cd stress (Figure 2). Cd stress considerably induced the MDA and H_2_O_2_ contents and their maximum increase was recorded under 20 mg kg^−1^ Cd stress (Figure 2). Conversely, Tre application (30 mM) markedly reduced MDA and H_2_O_2_ accumulation, indicating its important role in ameliorating Cd stress (Figure 2).

### 3.5. Total Soluble Proteins and Free Amino Acids

**Figure 3:**
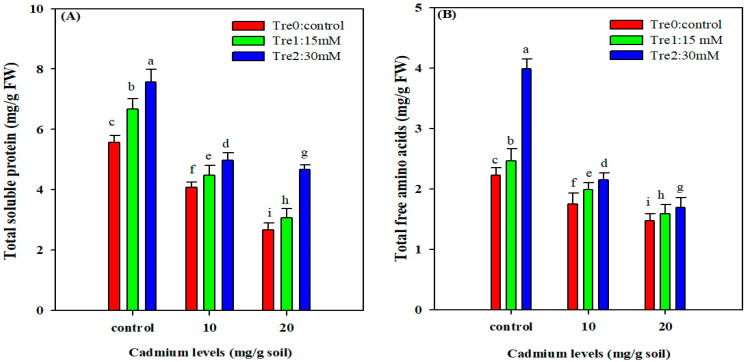
Effect of foliar application of Tre on TSP (**A**); and FAA (**B**) of mung bean plants grown under Cd stress. The values given in vertical bars are showing the mean of three replicates with ± S.E and the different letters above the bars under the same treatment show the significant differences at *p* < 0.05.

The TSP and FAA showed a marked reduction with an increased in the Cd stress; however, Tre application showed a marked improvement in TSP and FAA under both Cd stress and normal conditions (Figure 3). The TSP was reduced by 46% and 89% whereas FAA was decreased by 51% and 35% under Cd (10 and 20 mg kg^−1^). The foliar supplementation of Tre (30 mM) significantly enhanced the TSP and FAA contents as compared to control and foliar spray of 15 mM Tre under normal and Cd stress conditions (Figure 3).

### 3.6. Antioxidant Enzymes Activities

**Figure 4:**
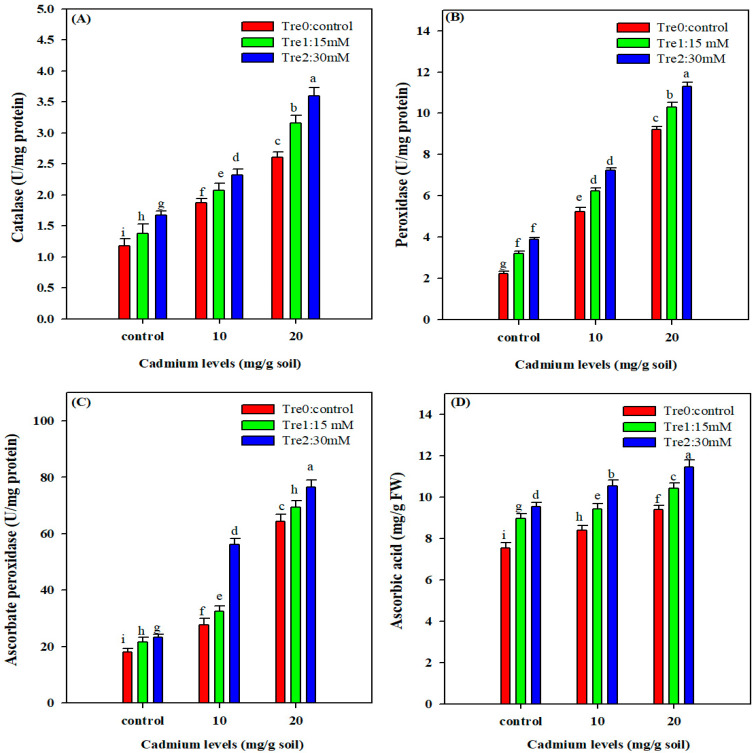
Effect of foliar spray of Tre on activities of CAT (**A**), POD (**B**), APX (**C**) and AsA (**D**) in mung bean plants grown under Cd stress. The values given in vertical bars are showing the mean of three replicates with ± S.E and the different letters above the bars under the same treatment show the significant differences at *p* < 0.05.

The results demonstrated that the activities of antioxidant enzymes (CAT, POD, APX and AsA) were significantly enhanced under Cd stress (Figure 4). Interestingly, Tre application further enhanced the antioxidant enzymes’ activities. The foliar spray of Tre (30 mM) increased CAT, POD, APX and AsA activities under both the Cd stress levels (10 and 20 mg kg^−1^) (Figure 4).

### 3.7. Cd Concentration in Different Plant Organs

**Figure 5:**
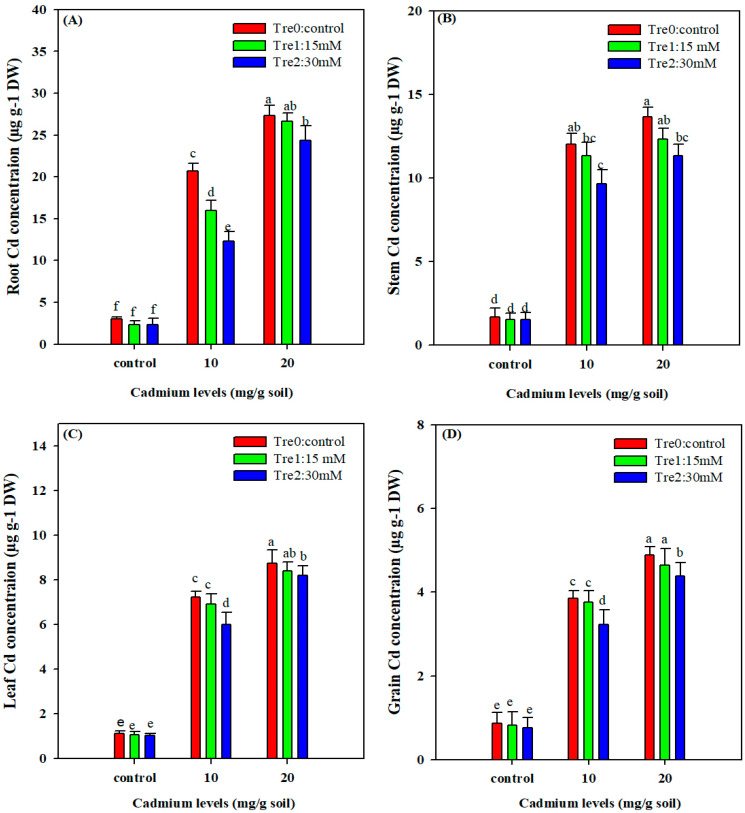
Effect of foliar spray of Tre on Cd concentration in the root (**A**); stem (**B**); leaf (**C**); and grain (**D**). Cd contents of mung bean plant grown under Cd stress. The values given in vertical bars are showing the mean of three replicates with ± S.E and the different letters above the bars under the same treatment show the significant differences at *p* < 0.05.

The concentration of Cd in the tested plant organs was significantly increased under Cd. On the other hand, Tre application significantly decreased the Cd accretion in plant organs (Figure 5). The maximum Cd concentration in roots and stems was recorded in the highest Cd stress (20 mg kg^−1^) without Tre application, and the lowest Cd concentration in root and stem was recorded in control (no Cd) with highest Tre application (30 mM) (Figure 5). Likewise, in the leaves and grains, the maximum Cd concentration was recorded under the 20 mg kg^−1^ Cd stress (Figure 5). Tre application (30 mM) significantly reduced Cd accumulation in mung bean seeds and leaves (Figure 5).

### 3.8. Yield Components

Cd stress significantly reduced the yield components of mung bean plants. However, Tre ameliorated the negative effects of Cd stress and improved yield of mung bean plants under normal and Cd stress conditions (Table 3). The longer pods (12.20 cm) with more grains (12.67) were recorded under no Cd stress with Tre application of 30 mM, but the shorter pods (8.02 cm) with minimum grains (8.33) were noted under Cd stress (20 mg kg^−1^) without Tre application (Table 3). Cd stress levels also significantly reduced the grain weight and grain yield (Table 3) and the maximum reduction was noted under 20 mg kg^−1^ Cd stress conditions. Conversely Tre supply (30 mM) markedly improved the 100-grain weight and grain yield under Cd stress (Table 3).

### 3.9. Pearson’s Correlation Analysis

**Figure 6:**
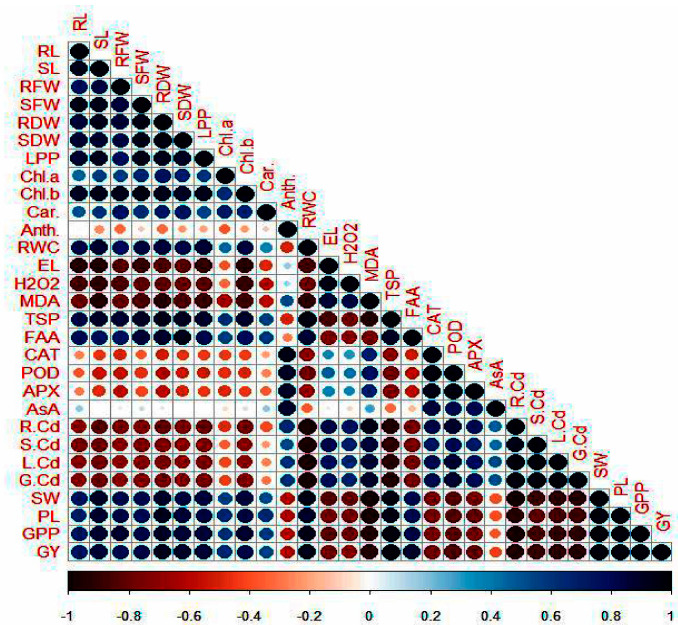
Pearson’s correlation between the studied traits of mung bean. Anth: anthocyanin; APX: ascorbate peroxidase; AsA: ascorbic acid; Chl a: chlorophyll a; Chl b: chlorophyll b; Car: carotenoid; CAT: catalase; EL: electrolyte leakage; FAA: free amino acid; GPP: grains/pod; G.Cd: grain Cd concentration; GY: grain yield; H_2_O_2_: hydrogen peroxide; LPP: leavers per plant; L.Cd: leaf Cd concentration; MDA: malondialdehyde; PL; pod length; POD: peroxidase; RFW: root fresh weight; RL: root length; RDW: root dry weight; RWC: relative water content; R.Cd: root Cd concentration; SL: shoot length; SFW: shoot fresh weight; SDW: shoot dry weight; S.Cd: stem Cd concentration; SW: 100 seed weight; TSP: total soluble protein.

The data of diverse traits recorded for soil-grown plants were subjected to Pearson’s correlation analysis to determine the relationship among different parameters (Figure 6). The results indicated that Cd concentration had positive linking with EL, MDA and H_2_O_2_ accumulation while it had negative correlations with growth, yield, TSA, FAA and RWC and photosynthetic pigments. Moreover, a strong positive correlation was also noted between Cd concentration and antioxidant activities. Conversely, Tre was positively correlated with biomass, yield, photosynthetic pigments, TSP, FAA, RWC and anti-oxidant enzymes while it had negative correlations with EL, MDA and H_2_O_2_ and Cd accumulation in different plants’ organs.

## 4. Discussion

Heavy metals induced serious alterations in the physiological and biochemical processes, growth, photosynthetic pigments and antioxidant defenses of plants [52]. Heavy metals’ pollution is a serious concern to plant growth and human health; therefore, proper technologies must be adopted to control the heavy metal pollution in order to ensure better crop productivity and human health. Cadmium is a toxic and easily absorbed metal by plants and causes negative impacts on plant growth and development [53]. In the present study, Cd stress induced oxidative stress in mung bean plants, resulting in significant reductions in growth and biomass production [54]. Cd stress also inhibited the nutrients acquisition and photosynthesis which reduced the assimilates’ production and thereby caused significant reductions in growth [55,56]. On the other hand, Tre supply significantly increased the growth and biomass production. This increase in plant growth and biomass production by Tre could be attributed to the Tre-mediated increases in RWC and photosynthetic performance and reductions in MDA and H_2_O_2_ accumulation due to the significant increases in antioxidant activities. We also hypothesized that Tre might also reduce the Cd uptake by forming Tre-Cd complexes and therefore improved the plant growth and biomass production. Trehalose possesses a relatively low surface potential and it readily interacts with Cd, resulting in reduced Cd uptake and improved growth and biomass [57].

Photosynthesis is an imperative plant physiological process that could be severely inhibited by Cd stress, based on the Cd doses applied [10,58]. In the present study, Cd stress markedly reduced the photosynthetic pigments. This was in line with the previous reports which revealed that Cd stress could reduce the absorption of Mg, Fe, K and P from soil and reduce the formation of leaf porphyrin rings, resulting in a marked reduction in chlorophyll synthesis [59] owing to the fact that Mg is a building block in the formation of chlorophyll contents. However, Tre supplementation significantly increased the synthesis of photosynthetic pigments, indicating that Trehalose supply might protect the membrane integrity and photosynthetic apparatus from oxidative stress via the reduction of the activity of chlorophyll degrading enzymes under stress conditions [60].

In the present study, RWC was significantly reduced under Cd stress. This could be attributed to the fact that Cd stress could reduce the osmotic potential and water uptake and subsequently reduce RWC [61]. Moreover, ABA-mediated stomatal closure due to Cd stress could reduce the transpiration pull and led to limited water uptake by plant roots and entails lower RWC in mung bean plants. On the other hand, Tre supply significantly improved RWC under both normal and Cd stress conditions, indicating that Tre supplementation protects the membrane and osmotic potential and scavenges the ROS formation and, therefore, could improve the water retention in plant organs under stress conditions [62]. Moreover, it is also hypothesized that Tre might reduce the ABA-mediated stomata closure and enhance the water uptake and RWC under Cd stress. In the present study, EL, H_2_O_2_ and MDA also showed a significant increase with increasing the Cd stress. Such increase is possibly due to alterations in membrane structure and changes in cellular homeostasis. However, Tre supply reduced the electrolyte leakage which might be due to the maintenance of membrane integrity and scavenging of ROS due to the increases in antioxidant activities [63]. The exposure of plants to Cd stress induced the production of ROS which damages the membrane [5]. However, in the present study, Tre supplied by foliar application markedly reduced the oxidative stress by decreasing the production of H_2_O_2_ and MDA contents under Cd stress. The reduction in MDA was due to the decrease in membrane damage. Tre supplementation might maintain the appreciable levels of K^+^ and Ca^2+^ under Cd which protect the membranes from oxidative stress and thereby could reduce MDA and EL under stress conditions. Tre might be participating in stress signaling and triggers osmolytes’ accumulation and antioxidant activities and therefore reduces the ROS production under Cd stress.

The exposure of plants to heavy metals might increase the activity of anti-oxidants; however, the very high metal concentration might destroy the protective enzymes system, and thereby decreases the antioxidant activities [64]. The antioxidant enzymes (CAT, POD, and APX) activity was markedly increased under Cd stress. Furthermore, Tre application further increased the antioxidant enzyme activities. Similarly, Tre supply also enhanced the activity of antioxidants which play a crucial role in counteracting the heavy metal-induced stress [65]. Therefore, it can also be concluded that Tre directly mediates the signaling transduction network or indirectly improves osmolytes’ accumulations which trigger antioxidant activities and encounter Cd-induced toxic effects.

Proteins play multipurpose functions in plants; however, their activity is degraded under different metal stresses. In the current study, TSP and FAA were significantly reduced under Cd stress. This could be due to the fact that Cd stress toxicity increases protease activity, which triggers the degradation of proteins [66], resulting in reduced protein accumulation under Cd stress. Cd toxicity disrupts the metabolism of amino acids, and changes in amino acid level could play a crucial role in plant responsiveness to Cd stress [67,68]. Tre supply increased the concentrations of FAA and TSP under Cd stress. This increase could be attributed to the ability of Tre to stabilize proteins and dehydrated enzymes involved in protein and amino acid synthesis. Plant species varied in their ability to absorb Cd from the soil and transport it into different plant organs [69]. The quantity of Cd absorbed by plants and its translocation to shoots depend on its bonding with the extracellular matrix, roots efflux, complexion within cells and transport efficiency [70]. In the current study, it was noted that the roots accumulated more Cd as compared to stems, leaves and grains, which could be attributed to the fact that the roots came into direct contact with Cd. However, Tre supplementation had an inhibitory effect on Cd accumulation and significantly reduced Cd accumulation in mung bean plant organs. A possible reason for this reduction could be that Tre acts as barrier to the Cd uptake in plant root which, therefore, tends to reduce the Cd transportation and accumulation in upper plant organs. Furthermore, Tre might also form complexes with Cd and reduce its uptake by plant roots and therefore reduce the Cd accumulation.

Cd toxicity significantly reduced the yield traits of mung bean. This reduction in yield could be attributed to the elevated oxidative stress levels, accumulation of MDA and H_2_O_2_, Cd uptake and pronounced decreases in photosynthetic pigments, RWC, and protein and amino acids’ accumulation. Tre supplementation considerably increased the yield traits under normal and Cd stress conditions. Tre protected the cell membrane and proteins and ensured better chlorophyll synthesis, leaf RWC, TSP and FAA accumulation, antioxidant activities, this markedly improved the yield traits [71].

## 5. Conclusions

Tre supplementation significantly enhanced Cd stress tolerance of mung bean crop. Cd stress markedly reduced the mung bean growth and yield which was associated with significant reduction in photosynthetic pigments and increases in ROS production and MDA accumulation. Nonetheless, Tre supply markedly improved mung bean yield by decreasing the Cd uptake, improving photosynthetic pigments, TSP and FAA and scavenging ROS by triggering the anti-oxidant enzymes. Therefore, these findings concluded that Tre supply could strengthen mung bean anti-oxidant defenses and ameliorate the Cd-induced deleterious effects on mung bean growth and yield.

## Figures and Tables

**Table 1:** Effect of Tre supply on the growth and biomass traits of mung bean grown in soil contaminated with Cd.

Treatments		Root Length (cm)	Shoot Length (cm)	Root Fresh Weight (g)	Shoot Fresh Weight (g)	Root Dry Weight (g)	Shoot Dry Weight (g)	Leaves Per Plant
Cd_0_	Tre_0_	4.89 ± 0.029 ^cd^	25.40 ± 0.036 ^c^	0.76 ± 0.004 ^c^	5.23 ± 0.111 ^de^	0.27 ± 0.003 ^c^	0.71 ± 0.007 ^c^	11.0 ± 0.559 ^ab^
Cd_0_	Tre_1_	5.90 ± 0.158 ^b^	27.13 ± 0.059 ^ab^	1.16 ± 0.044 ^a^	6.96 ± 0.076 ^b^	0.34 ± 0.007 ^b^	0.83 ± 0.005 ^b^	12.0 ± 0.211 ^ab^
Cd_0_	Tre_2_	6.67 ± 0.075 ^a^	28.10 ± 0.073 ^a^	1.13 ± 0.021 ^a^	7.67 ± 0.055 ^a^	0.41 ± 0.004 ^a^	1.03 ± 0.020 ^a^	13.0 ± 0.366 ^a^
Cd_1_	Tre_0_	3.43 ± 0.045 ^f^	22.29 ± 0.124 ^e^	0.63 ± 0.004 ^d^	4.73 ± 0.021 ^f^	0.26 ± 0.004 ^c^	0.69 ± 0.005 ^c^	11.0 ± 0.422 ^bc^
Cd_1_	Tre_1_	4.13 ± 0.049 ^e^	23.72 ± 0.039 ^d^	1.00 ± 0.013 ^b^	4.93 ± 0.055 ^ef^	0.26 ± 0.004 ^c^	0.73 ± 0.007 ^c^	11.0 ± 0.559 ^bc^
Cd_1_	Tre_2_	5.33 ± 0.042 ^c^	26.57 ± 0.055 ^bc^	0.84 ± 0.004 ^c^	6.17 ± 0.042 ^c^	0.32 ± 0.007 ^b^	0.88 ± 0.009 ^b^	10.0 ± 0.366 ^cd^
Cd_2_	Tre_0_	3.17 ± 0.091 ^f^	19.20 ± 0.036 ^f^	0.49 ± 0.004 ^e^	3.83 ± 0.092 ^g^	0.15 ± 0.004 ^e^	0.57 ± 0.002 ^e^	9.0 ± 0.634 ^d^
Cd_2_	Tre_1_	4.67 ± 0.046 ^d^	22.97 ± 0.046 ^de^	0.60 ± 0.005 ^d^	4.83 ± 0.057 ^ef^	0.21 ± 0.004 ^d^	0.64 ± 0.005 ^d^	9.0 ± 0.332 ^d^
Cd_2_	Tre_2_	4.97 ± 0.062 ^cd^	24.13 ± 0.107 ^d^	0.77 ± 0.006 ^c^	5.56 ± 0.112 ^d^	0.25 ± 0.004 ^c^	0.70 ± 0.004 ^c^	10.0 ± 0.211 ^cd^

The values given in table show the mean of three replicates with ± S.E and different letters show the significant differences at *p* < 0.05. Cd_0_, Cd_1_ and Cd_2_ indicate 0, 10 and 20 mg of Cd kg^−1^ of soil and Tre_0_, Tre_1_ and Tre_2_ indicate 0, 15 and 30 mM Trehalose.

**Table 2:** Effect of Tre supply on the growth and biomass traits of mung bean grown in hydroponic culture under normal and Cd stress conditions.

Treatments		Root Length (cm)	Shoot Length (cm)	Root Fresh Weight (g)	Shoot Fresh Weight (g)	Root Dry Weight (g)	Shoot Dry Weight (g)
Cd_0_	Tre_0_	3.55 ± 0.048 ^e^	20.56 ± 0.077 ^c^	0.61 ± 0.005 ^c^	4.71 ± 0.087 ^d^	0.21 ± 0.004 ^d^	0.53 ± 0.006 ^d^
Cd_0_	Tre_1_	4.88 ± 0.097 ^b^	21.68 ± 0.062 ^b^	0.80 ± 0.053 ^b^	5.11 ± 0.082 ^c^	0.28 ± 0.008 ^b^	0.63 ± 0.007 ^c^
Cd_0_	Tre_2_	5.79 ± 0.112 ^a^	22.52 ± 0.054 ^a^	0.96 ± 0.016 ^a^	6.15 ± 0.075 ^a^	0.35 ± 0.006 ^a^	0.91 ± 0.011 ^a^
Cd_1_	Tre_0_	3.06 ± 0.083 ^f^	17.11 ± 0.086 ^f^	0.44 ± 0.006 ^d^	3.61 ± 0.048 ^f^	0.17 ± 0.007 ^e^	0.45 ± 0.008 ^e^
Cd_1_	Tre_1_	3.95 ± 0.091 ^d^	18.42 ± 0.104 ^e^	0.62 ± 0.023 ^c^	4.62 ± 0.062 ^d^	0.24 ± 0.005 ^c^	0.53 ± 0.006 ^d^
Cd_1_	Tre_2_	4.42 ± 0.065 ^c^	19.51 ± 0.113 ^d^	0.79 ± 0.008 ^b^	5.58 ± 0.051 ^b^	0.29 ± 0.006 ^b^	0.69 ± 0.008 ^b^
Cd_2_	Tre_0_	2.81 ± 0.088 ^g^	14.01 ± 0.098 ^h^	0.31 ± 0.005 ^e^	3.01 ± 0.063 ^g^	0.12 ± 0.006 ^f^	0.43 ± 0.004 ^e^
Cd_2_	Tre_1_	3.56 ± 0.057 ^e^	15.89 ± 0.087 ^g^	0.46 ± 0.008 ^d^	4.19 ± 0.071 ^e^	0.17 ± 0.005 ^e^	0.61 ± 0.006 ^c^
Cd_2_	Tre_2_	4.34 ± 0.091 ^c^	17.08 ± 0.111 ^f^	0.60 ± 0.009 ^c^	5.07 ± 0.092 ^c^	0.21 ± 0.006 ^d^	0.63 ± 0.005 ^c^

The values given in the table show the mean of three replicates with ± S.E and different letters show the significant differences at *p* < 0.05. Cd_0_, Cd_1_ and Cd_2_ indicate 0, 200 and 400 μM of Cd and Tre_0_, Tre_1_ and Tre_2_ indicate 0, 15 and 30 mM Trehalose.

**Table 3:** Effect of trehalose supply on yield traits of mung bean crop under different levels of Cd stress.

Treatments		Pod Length (cm)	Grains/Pod	100 Seed Weight (g)	Grain Yield (g pot^−1^)
Cd_0_	Tre_0_	11.13 ± 0.14 ^bc^	11.33 ± 0.27 ^abc^	6.24 ± 0.034 ^b^	37.67 ± 1.29 ^bc^
Cd_0_	Tre_1_	11.46 ± 0.21 ^b^	12.33 ± 0.29 ^ab^	6.33 ± 0.053 ^b^	39.67 ± 1.49 ^ab^
Cd_0_	Tre_2_	12.20 ± 0.16 ^a^	12.67 ± 0.54 ^a^	6.65 ± 0.062 ^a^	44.00 ± 1.41 ^a^
Cd_1_	Tre_0_	10.20 ± 0.17 ^e^	9.67 d ± 0.53 ^de^	5.03 ± 0.030 ^e^	33.00 ± 1.25 ^d^
Cd_1_	Tre_1_	10.62 ± 0.12 ^de^	10.67 ± 0.27 ^bcd^	5.47 ± 0.110 ^d^	32.67 ± 1.18 ^d^
Cd_1_	Tre_2_	10.87 ± 0.11 ^cd^	11.00 ± 0.47 ^bcd^	5.92 ± 0.072 ^c^	35.00 ± 0.94 ^cd^
Cd_2_	Tre_0_	8.02 ± 0.03 ^g^	8.33 ± 0.29 ^f^	4.13 ± 0.064 ^g^	25.67 ± 0.98 ^f^
Cd_2_	Tre_1_	8.60 ± 0.14 ^f^	8.33 ± 0.30 ^f^	4.44 ± 0.047 ^f^	26.67 ± 0.72 ^e^
Cd_2_	Tre_2_	9.07 ± 0.07 ^f^	9.00 ± 0.48 ^e^	4.61 ± 0.072 ^f^	28.00 ± 1.69 ^e^

The values given in the table are showing the mean of three replicates with ± S.E and different letters showing the significant differences at *p* < 0.05. Cd_0_, Cd_1_ and Cd_2_ showing 0, 10 and 20 mg of Cd kg^−1^ of soil and Tre_0_, Tre_1_ and Tre_2_ indicating trehalose application at different levels, i.e., control (0 mM), 15 mM and 30 mM.

## Data Availability

All the data supporting this study are included in the article.

## References

[1] Hassan M.U., Chattha M.U., Khan I., Chattha M.B., Aamer M., Nawaz M., Ali A., Khan M.A.U., Khan T.A. (2019). Nickel toxicity in plants: Reasons, toxic effects, tolerance mechanisms, and remediation possibilities—A review. Environ. Sci. Poll. Res..

[2] Zhao H., Guan J., Liang Q., Zhang X., Hu H., Zhang J. (2021). Effects of cadmium stress on growth and physiological characteristics of sassafras seedlings. Sci. Rep..

[3] Ali B., Qian P., Jin R., Ali S., Khan M., Aziz R., Tian T., Zhou W. (2014). Physiological and ultra-structural changes in Brassica napus seedlings induced by cadmium stress. Biol. Plant..

[4] Tang Y., Xie Y., Sun G., Tan H., Lin L., Li H., Liao M.A., Wang Z., Lv X., Liang D. (2018). Cadmium-accumulator straw application alleviates cadmium stress of lettuce (*Lactuca sativa*) by promoting photosynthetic activity and antioxidative enzyme activities. Environ. Sci. Poll. Res..

[5] Aamer M., Muhammad U.H., Li Z., Abid A., Su Q., Liu Y., Adnan R., Muhammad A.U.K., Tahir A.K., Huang G. (2018). Foliar application of glycinebetaine (GB) alleviates the cadmium (Cd) toxicity in spinach through reducing Cd uptake and improving the activity of anti-oxidant system. Appl. Ecol. Environ. Res..

[6] Chattha M.U., Arif W., Khan I., Soufan W., Bilal M.C., Hassan M.U., Ullah N., Sabagh A.E., Qari S.H. (2021). Mitigation of cadmium induced oxidative stress by using organic amendments to improve the growth and yield of mash beans [*Vigna mungo* (L.)]. Agronomy.

[7] Rasheed A., Fahad S., Aamer M., Hassan M.U., Tahir M.M., Wu Z. (2020). Role of genetic factors in regulating cadmium uptake, transport and accumulation mechanisms and quantitative trait loci mapping in rice. a review. Appl. Ecol. Environ. Res..

[8] Hussain S., Irfan M., Sattar A., Hussain S., Ullah S., Abbas T., Ur-Rehman H., Nawaz F., Al-Hashimi A., Elshikh M.S. (2022). Alleviation of cadmium stress in wheat through the combined application of boron and biochar via regulating morpho-physiological and antioxidant defense mechanisms. Agronomy.

[9] Chen H.C., Zhang S.L., Wu K.J., Li R., He X.R., He D.N., Huang C., Wei H. (2020). The effects of exogenous organic acids on the growth, photosynthesis and cellular ultrastructure of *Salix variegata* Franch. Under Cd stress. Ecotoxicol. Environ. Saf..

[10] Imran K., Seleiman M.F., Chattha M.U., Jalal R.S., Mahmood F., Hassan F.A., Izzet W., Alhammad B.A., Rana R., Hassan M.U. (2021). Enhancing antioxidant defense system of mung bean with a salicylic acid exogenous application to mitigate cadmium toxicity. Not. Bot. Horti Agrobot. Cluj-Napoca.

[11] Rizwan M., Ali S., Abbas T., Zia-ur-Rehman M., Hannan F., Keller C., Al-Wabel M.I., Ok Y.S. (2016). Cadmium minimization in wheat: A critical review. Ecotox. Environ. Saf..

[12] Zou J., Wang G., Ji J., Wang J., Wu H., Ou Y., Li B. (2017). Transcriptional, physiological and cytological analysis validated the roles of some key genes linked Cd stress in *Salix matsudana* Koidz. Environ. Exper. Bot..

[13] Hassan M.U., Aamer M., Chattha M.U., Haiying T., Shahzad B., Barbanti L., Nawaz M., Rasheed A., Afzal A., Liu Y. (2020). The critical role of zinc in plants facing the drought stress. Agriculture.

[14] Hassan M.U., Chattha M.U., Khan I., Chattha M.B., Barbanti L., Aamer M., Iqbal M.M., Nawaz M., Mahmood A., Ali A. (2021). Heat stress in cultivated plants: Nature, impact, mechanisms, and mitigation strategies—A review. Plant Biosyst..

[15] Dustgeer Z., Seleiman M.F., Imran K., Chattha M.U., Alhammad B.A., Jalal R.S., Refay Y., Hassan M.U. (2021). Glycine-betaine induced salinity tolerance in maize by regulating the physiological attributes, antioxidant defense system and ionic homeostasis. Not. Bot. Horti Agrobot..

[16] Batool M., El-Badri A.M., Hassan M.U., Haiyun Y., Chunyun W., Zhenkun Y., Jie K., Wang B., Zhou G. (2022). Drought stress in Brassica napus: Effects, tolerance mechanisms, and management strategies. J. Plant Growth Reg..

[17] Seleiman M.F., Aslam M.T., Alhammad B.A., Hassan M.U., Maqbool R., Chattha M.U., Khan I., Gitari H.I., Uslu O.S., Rana R. (2022). Salinity stress in wheat: Effects, mechanisms and management strategies. Phyton.

[18] Sultan I., Khan I., Chattha M.U., Hassan M.U., Barbanti L., Calone R., Ali M., Majid S., Ghani M.A., Batool M. (2021). Improved salinity tolerance in early growth stage of maize through salicylic acid foliar application. Ital. J. Agron..

[19] Li S., Yu J., Zhu M., Zhao F., Luan S. (2012). Cadmium impairs ion homeostasis by altering K^+^ and Ca^2+^ channel activities in rice root hair cells. Plant Cell Environ..

[20] Xiong T., Dumat C., Pierart A., Shahid M., Kang Y., Li N., Bertoni G., Laplanche C. (2016). Measurement of metal bioaccessibility in vegetables to improve human exposure assessments: Field study of soil–plant–atmosphere transfers in urban areas, South China. Environ. Geochem. Health.

[21] Qu J.H., Meng X.L., Jiang X.Y., You H., Wang P., Ye X.Q. (2018). Enhanced removal of Cd (II) from water using sulfur-functionalized rice husk: Characterization, adsorptive performance and mechanism exploration. J. Clean. Prod..

[22] Subašić M., Šamec D., Selović A., Karalija E. (2022). Phytoremediation of Cadmium Polluted Soils: Current Status and Approaches for Enhancing. Soil Syst..

[23] Wang K., Li F., Gao M., Huang Y., Song Z. (2020). Mechanisms of trehalose-mediated mitigation of Cd toxicity in rice seedlings. J. Clean. Prod..

[24] Gharaei A., Hoseini S.S.A., Karimi M., Pourjavad E., Amjadian A. (2019). An integrated stochastic EPQ model under quality and green policies: Generalized cross decomposition under the separability approach. Int. J. Syst. Sci. Oper. Logist..

[25] Yang Y., Ge Y., Tu P., Zeng H., Zhou X., Zou D., Wang K., Zeng Q. (2019). Phytoextraction of Cd from a contaminated soil by tobacco and safe use of its metal-enriched biomass. J. Hazard Mater..

[26] Zulfiqar F., Ashraf M., Siddique K.H. (2022). Role of glycine betaine in the thermotolerance of plants. Agronomy.

[27] Zulfiqar F., Chen J., Finnegan P.M., Nafees M., Younis A., Shaukat N., Latif N., Abideen Z., Zaid A., Raza A. (2021). Foliar application of trehalose or 5-aminolevulinic acid improves photosynthesis and biomass production in drought stressed Alpinia zerumbet. Agriculture.

[28] Shoa J., Wu W., Fahd R., Hassan M., Kai H., Masood I.W., Tasahil S.A., Muhammad A., Hu Q., Huang G. (2022). Trehalose induced drought tolerance in plants: Physiological and molecular responses. Not. Bot. Horti Agrobot..

[29] Duman F., Aksoy A., Aydin Z., Temizgul R. (2010). Effects of exogenous glycinebetaine and trehalose on cadmium accumulation and biological responses of an aquatic plant (*Lemna gibba* L). Water Air Soil Pollut..

[30] Ali Q., Ashraf M. (2011). Induction of drought tolerance in maize (*Zea mays* L.) due to exogenous application of trehalose: Growth, photosynthesis, water relations and oxidative defence mechanism. J. Agron. Crop Sci..

[31] Ponnu J., Wahl V., Schmid M. (2011). Trehalose-6-phosphate: Connecting plant metabolism and development. Front. Plant Sci..

[32] Blazquez M.A., Santos E., Floras C.L., Martinez-Zapater J.M., Salinas J., Gancedo C. (1998). Isolation and molecular characterization of the Arabidopsis TPS1 gene, encoding trehalose-6-phosphate synthase. Plant J..

[33] Zentella R., Mascorro-Gallardo J.O., Van Dijck P., Folch-Mallol J., Bonini B., Van-Vaeck C., Gaxiola R., Covarrubias A.A., Nieto-Sotelo J., Thevelein J.M. (1999). A *Selaginella lepidophylla* trehalose-6-phosphate synthase complements growth and stress-tolerance defects in a yeasttps1 mutant. Plant Physiol..

[34] Vogel G., Aeschbacher R.A., Müller J., Boller T., Wiemken A. (1998). Trehalose-6-phosphate phosphatases from Arabidopsis thaliana: Identification by functional complementation of the yeast tps2 mutant. Plant J..

[35] Fernandez O., Béthencourt L., Quero A., Sangwan R.S., Clément C. (2010). Trehalose and plant stress responses: Friend or foe?. Trends Plant Sci..

[36] Sadak M.S. (2019). Physiological role of trehalose on enhancing salinity tolerance of wheat plant. Bull. Nat. Res. Cent..

[37] Bae H., Herman E., Bailey B., Bae H.J., Sicher R. (2005). Exogenous trehalose alters Arabidopsis transcripts involved in cell wall modification, abiotic stress, nitrogen metabolism, and plant defense. Physiol. Plant.

[38] Alemu I.D. (2016). General characteristics and genetic improvement status of mung bean (*Vigna radiata* L.) in Ethiopia. Intern. J. Agric. Inno. Res..

[39] Muhammad U.C., Muhammad U.H., Imran K., Muhammad B.C., Imran A., Muhammad F., Mina K. (2017). Effect of different nitrogen and phosphorus fertilizer levels in combination with nitrogen and phosphorus solubilizing inoculants on the growth and yield of mung bean. Pak. J. Life Soc. Sci..

[40] Arnon D.I. (1949). Copper enzymes in isolated chloroplasts, polyphenoxidase in *Beta vulgaris*. Plant Physiol..

[41] Mostofa M.G., Fujita M. (2013). Salicylic acid alleviates copper toxicity in rice (*Oryza sativa* L.) seedlings by up-regulating antioxidative and glyoxalase systems. Ecotoxicology.

[42] Cakmak I., Horst W.J. (1991). Effect of aluminium on lipid peroxidation, superoxide dismutase, catalase, and peroxidase activities in root tips of soybean (*Glycine max*). Physiol. Plant..

[43] Velikova V., Yordanov I., Edreva A. (2000). Oxidative stress and some antioxidant systems in acid rain-treated bean plants. Protective role of exogenous polyamines. Plant Sci..

[44] Bradford M.M. (1976). A rapid and sensitive method for the quantitation of microgram quantities of protein utilizing the principle of protein-dye binding. Anal. Biochem..

[45] Vanslyke D.D., Macfadyen D.A., Hamilton P.B. (1943). The gasometric determination of amino acids in mine by the ninhydrin-carbon dioxide method. J. Biol. Chem..

[46] Chance B., Maehly A.C. (1955). Assay of catalases and peroxidases. Methods Enzymol..

[47] Guan J.S., Haggarty S.J., Giacometti E., Dannenberg J.H., Joseph N., Gao J., Nieland T.J., Zhou Y., Wang X., Mazitschek R. (2009). HDAC2 negatively regulates memory formation and synaptic plasticity. Nature.

[48] Nakano Y., Asada K. (1987). Purification of ascorbate peroxidase in spinach chloroplasts; Its inactivation in ascorbate-depleted medium and reactivation by mono-dehydroascorbate radical. Plant Cell Physiol..

[49] Mukherjee S.P., Choudhuri M.A. (1983). Implications of water stree-induced changes in the leaves of indigenous ascorbic acid and hydrogen peroxide in Vigna seedlings. Physiol. Plant..

[50] Jones J.B., Case V.W. (1990). Sampling, handling, and analyzing plant tissue samples. Soil Test. Plant Anal..

[51] Steel R.G.D., Torrie J.H., Dicky D.A. (1997). Principles and Procedures of Statistics: A Biometrical Approach.

[52] Cao F., Wang R., Cheng W., Zeng F., Ahmed I.M., Hu X., Zhang G., Wu F. (2014). Genotypic and environmental variation in cadmium, chromium, lead and copper in rice and approaches for reducing the accumulation. Sci. Total Environ..

[53] Saidi I., Ayouni M., Dhieb A., Chtourou Y., Chaïbi D.W. (2013). Oxidative damages induced by short-term exposure to cadmium in bean plants: Protective role of salicylic acid. S. Afr. J. Bot..

[54] Wu F.Z., Yang W.Q., Zhang J., Zhou L.Q. (2010). Effects of cadmium stress on growth and nutrient accumulation, distribution and utilization in *Osmanthus fragrans* var. thunbergii. Chin. J. Plant Ecol..

[55] Cengiz K., Nudrat A., Akram M., Ashraf M., Nasser A., Parvaiz A. (2020). Exogenously supplied silicon (Si) improves cadmium tolerance in pepper (*Capsicum annuum* L.) by upregulating the synthesis of nitric oxide and hydrogen sulfide. J. Biotechnol..

[56] Wang H., Zhao S.C., Xia W.J. (2008). Effects of cadmium stress at different concentrations on photosynthesis, lipid peroxidation and antioxidant enzyme activities in maize seedlings. J. Plant Nutr. Fert..

[57] Dong L.B., Yu D., Lin X.T., Wang B., Pan L. (2020). Improving expression of thermostable trehalase from *Myceliophthora sepedonium* in Aspergillus Niger mediated by the CRISPR/Cas9 tool and its purification, characterization. Protein Expr. Purif..

[58] Zulfiqar U., Ayub A., Hussain S., Waraich E.A., El-Esawi M.A., Ishfaq M., Ahmad M., Ali N., Maqsood M.F. (2022). Cadmium Toxicity in Plants: Recent Progress on Morpho-physiological Effects and Remediation Strategies. J. Soil Sci. Plant Nutr..

[59] Chen X., Pu G., Huang Y., Mo L. (2019). Effects of thallium and cadmium stress on growth and photosynthetic characteristics of *Arundo donax*. Guangxi Zhiwu/Guihaia.

[60] Kosar F., Akram N.A., Ashraf M., Sadiq M., Al-Qurainy F. (2018). Trehalose-induced improvement in growth, photosynthetic characteristics and levels of some key osmoprotectants in sunflower (*Helianthus annuus* L.) under drought stress. Pak. J. Bot..

[61] Qin J., Dong W.Y., He K.N., Yu Y., Tan G.D., Han L., Dong M., Zhang Y.Y., Zhang D., Li A.Z. (2010). NaCl salinity-induced changes in water status, ion contents and photosynthetic properties of *Shepherdia argentea* (Pursh) Nutt. seedlings. Plant Soil Environ..

[62] Zeid I.M. (2009). Effect of arginine and urea on polyamines content and growth of bean under salinity stress. Acta Physiol. Plant.

[63] Zulfiqar F., Chen J., Finnegan P.M., Younis A., Nafees M., Zorrig W., Hamed K.B. (2021). Application of trehalose and salicylic acid mitigates drought stress in sweet basil and improves plant growth. Plants.

[64] Chaâbene Z., Rorat A., Hakim I.R., Bernard F., Douglas G.C., Elleuch A., Vandenbulcke F., Mejdoub H. (2018). Insight into the expression variation of metal-responsive genes in the seedling of date palm (*Phoenix dactylifera*). Chemosphere.

[65] Sharma A., Shahzad B., Rehman A., Bhardwaj R., Landi M., Zheng B. (2019). Response of Phenylpropanoid Pathway and the Role of Polyphenols in Plants under Abiotic Stress. Molecules.

[66] Palma J.M., Sandalio L.M., Corpas F.J., Romero-Puertas M.C., McCarthy I., Luis A. (2002). Plant proteases, protein degradation, and oxidative stress: Role of peroxisomes. Plant Physiol. Biochem..

[67] Zemanová V., Pavlík M., Pavlíková D., Tlustoš P. (2014). The significance of methionine, histidine and tryptophan in plant responses and adaptation to cadmium stress. Plant Soil Environ..

[68] Zoghlami L.B., Djebali W., Abbes Z., Hediji H., Maucourt M., Moing A., Brouquisse R., Chaibi W. (2011). Metabolite modifications in Solanum lycopersicum roots and leaves under cadmium stress. Afr. J. Biotechnol..

[69] Metwally A., Safronova V.I., Belimov A.A., Dietz K.J. (2005). Genotypic variation of the response to cadmium toxicity in *Pisum sativum* L.. J. Exp. Bot..

[70] Akhter M., Macfie S. (2012). Species-specific relationship between transpiration and cadmium translocation in lettuce, barley and radish. J. Plant Stud..

[71] Joshi R., Sahoo K.K., Singh A.K., Anwar K., Pundir P., Gautam R.K., Krishnamurthy S.L., Sopory S.K., Pareek A., Singla-Pareek S.L. (2020). Enhancing trehalose biosynthesis improves yield potential in marker-free transgenic rice under drought, saline, and sodic conditions. J. Exp. Bot..

